# *In Vitro* Sealing Properties of Calcium-Enriched Mixture and Mineral Trioxide Aggregate Orifice Barriers during Intra-Coronal Bleaching

**DOI:** 10.22037/iej.2017.45

**Published:** 2017

**Authors:** Negar Moghadam, Amir Ardalan Abdollahi, Hoda Aghabalayi Fakhim, Zahra Borna

**Affiliations:** a *Department of Endodontics, Dental School, Tabriz University of Medical Sciences, Tabriz, Iran;*; b *Department of Endodontics, Dental School, West Azarbaijan, Urmia University of Medical Sciences, Urmia, Iran; *; c *Private Practice, Tehran, Iran*

**Keywords:** Calcium-Enriched Mixture Cement, Cervical Barrier, Intra-Coronal Bleaching, Mineral Trioxide Aggregate

## Abstract

**Introduction:**

This study aimed at evaluating the sealing properties of calcium-enriched mixture (CEM) compared to mineral trioxide aggregate (MTA) as a cervical barriers in intra-coronal bleaching.

**Methods and Materials:**

In this *in vitro* study, endodontic treatment was performed on 60 extracted human incisors and canines without canal calcification, caries, restorations, resorption or cracks. The teeth were then randomly divided into two experimental groups and two control groups (*n*=15). Then, CEM cement and MTA were applied as 3-mm intra-orifice barriers in the test groups; a mixture of sodium perborate and 30% hydrogen peroxide bleaching agents were placed within the pulp chamber for one week. Dye penetration method was used to evaluate the sealing ability of agents. Statistical analysis was performed using SPSS software. The Kendall coefficient was used to evaluate inter-observer agreement. The chi-squared test was used for statistical analysis.

**Results:**

The results showed that the penetration rates of CEM and MTA were the same as positive control group, with no significant differences (*P*=0.673 and *P*=0.408, respectively). However, there was a significant difference between the negative control group and CEM and MTA groups (*P*=0.001 for both groups). In addition, the sealing ability of MTA and CEM cement were not significantly different (*P*=0.682).

**Conclusion:**

During intra-coronal bleaching procedures CEM cement can be used as a cervical barrier with sealing properties comparable to that of MTA.

## Introduction

Root canal-treated teeth usually become discolored due to trauma or inadequate pulp tissue removal [1]. Tooth bleaching used for non-vital teeth could be applied as an inexpensive and conservative method in comparison to crown and veneer techniques [2]. 

Cervical root resorption is one of the main side effects of non-vital bleaching, which might be attributed to the penetration of bleaching agents into the periodontal space through defects at the cementoenamel junction (CEJ) and dentinal tubules, causing necrosis of the cementum, inflammation of the periodontium and subsequently root resorption [3-5]. In addition, secondary microleakage due to a lack of proper coronal seal has a critical role in endodontic treatment failure [6]. Therefore a cervical or coronal barrier has a critical role in preventing postoperative complications [3, 6]. Several dental materials, including intermediate restorative material (IRM), hydraulic filling materials (Cavit and Coltosol), composite resins, photo-activated temporary resin materials, zinc oxide-eugenol cement, zinc phosphate cement and glass-ionomers (GI), have been recommended as coronal barriers during bleaching techniques [7].

**Figure 1:**
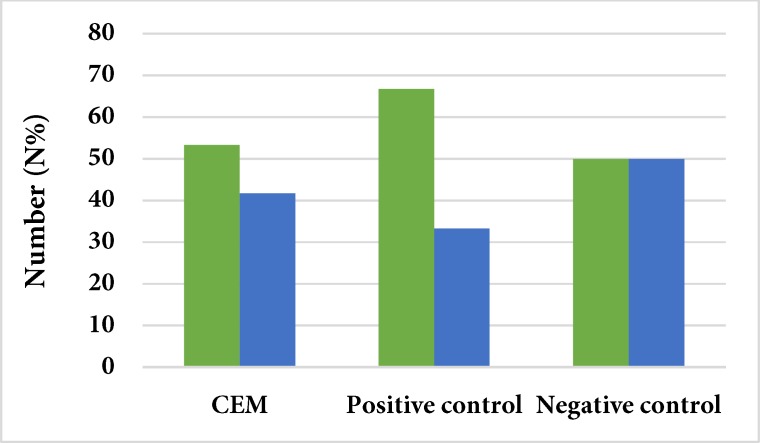
Comparison of the sealing ability of CEM with negative and positive controls

Mineral trioxide aggregate (MTA) was introduced to dentistry in the early 1990s as a biomaterial for endodontic applications [8]. MTA was first developed as a root-end filling material [9]. In addition, MTA has numerous clinical applications, including pulp capping, pulpotomy, treatment of internal root resorption, undeveloped apices (apexogenesis and apexification) and repair of root and furcation perforations [10, 11] and also as a coronal barrier [6]. MTA has some advantages, including favorable mechanical properties and setting expansion, resulting in an improvement in its sealing capacity and marginal adaptation, preventing microleakage [12].

Calcium-enriched mixture (CEM) is a hydrophilic cement formed by mixing a powder and a liquid. CEM exhibits appropriate characteristics that improve in the presence of water and humidity after its clinical application. CEM cement has the capacity to set and be used in the presence of moisture and exhibits good handling properties; in addition, it has a reasonable price [13-15]. CEM has exhibited better properties such as increased flow, similar sealing ability and reduced working time compared to MTA [14]. The sealing properties of CEM cement are comparable to those of MTA when it is used as a root-end filling material [16-18].

Since there is no study available to support the application of CEM cement as an appropriate coronal barrier agent in bleaching, this *in vitro* study was undertaken to evaluate the sealing properties of CEM cement compared to MTA as a cervical barrier in intra-coronal bleaching.

## Materials and Methods

In this *in vitro* study, 60 extracted single-rooted human maxillary incisors and canines without caries were selected. All the teeth were extracted because of periodontal disease. Approval was obtained from the Research and Ethics Committee of Tabriz University of Medical Sciences (Grant No: 22A). After confirmation of a straight non-calcified canal, the tooth roots were evaluated under a stereomicroscope and teeth with fractures or cracks, resorption and open apices were excluded. Following extraction, each tooth was stored in 3% chloramine-T solution at 4^°^C. Access cavity preparations were carried out with a tapered carbide bur. The working length was determined with a #15 K-Flexofile (Maillefer, Dentsply, Switzerland), 1 mm short of the apical foramen. All the root canals were instrumented in a crown-down manner. The coronal two-thirds of the canals were prepared with #4 and 3 Gates-Glidden drills (Maillefer, Dentsply, Switzerland), followed by the use of #40/0.10, #35/0.08 and #30/0.06 RaCe rotary files (FKG Dentaire, La-Chaux-de Fonds, Switzerland). Master apical file size of #35 K-Flexofile was established. Irrigation was carried out with 10 mL of 2.5% sodium hypochlorite during preparation and apical patency was preserved by using #10 K-Flexofile. Finally 5 mL of saline solution was used as the final irrigant. The root canals were dried with paper points and obturated with gutta-percha and AH-26 root canal sealer (Dentsply, De Trey, Konstanz, Germany) using the lateral condensation technique. 

Artificial defects were made along the CEJ. Cavities were made in the incisal area of teeth with a #1 high-speed drill in order to stabilize in experimental tubes. The root canals were sealed using temporary zinc oxide and hydrated zinc sulfate (Coltosol, Ariadent, Tehran, Iran). The samples were stored at 37^°^C and 100% humidity for one week. Peeso reamer #4 (Maillefer, Ballaigues, Switzerland) was used to remove the gutta-percha up to 3 mm below the CEJ in the palatal aspect. A periodontal probe was used to confirm the depth. The pulp chambers were irrigated with saline and dried with cotton pellets [19].

***Experimental groups***

Then 60 samples were randomly divided into two experimental and two control groups (*n*=15). The root canal orifices were sealed with MTA (Angelus, Londrina, Paraná, Brazil) and CEM cement (BioniqueDent, Tehran, Iran) in experimental groups 1 and 2, respectively, and wet cotton pellets were placed on the orifices. In group 3 (the negative control group), the canal orifices were left empty and in group 4 (the positive control group), the orifices were sealed with cyanoacrylate. The outer surface of the apical two-thirds and apices of the roots were covered with two layers of nail varnish. For bleaching procedure, a mixture of sodium perborate and 30% hydrogen peroxide was placed inside the pulp chamber for one week.

**Figure 2:**
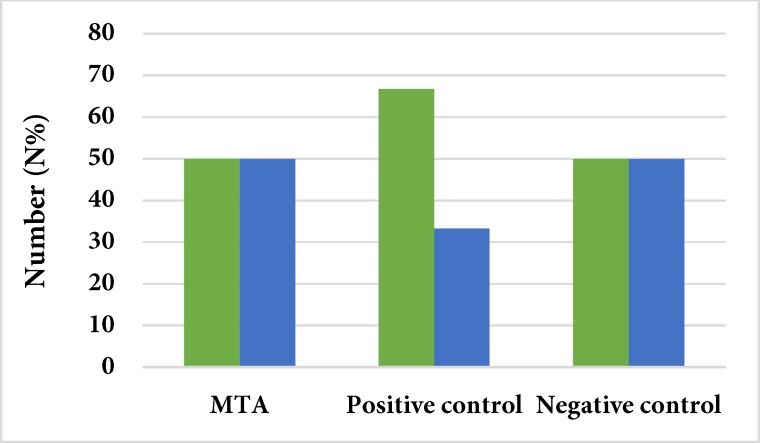
Comparison of the sealing ability of MTA with negative and positive controls

In this study, dye penetration method was used to evaluate the sealing ability of the agents. The samples were placed in experimental tubes containing potassium dichromate. Minimal contact with hydrogen peroxide could change the yellow color of potassium dichromate to blue. The samples were buried in potassium dichromate so that the root portions remained out of the liquid in order to prevent root leakage. The samples were retrieved from the liquid after 24 h and then the roots were sectioned in a mesiodistal direction into buccal and lingual halves using diamond disks 0.1 mm in diameter. The rate of dye penetration in the samples was evaluated under a stereomicroscope under ×10 and ×40 magnifications by two skilled and blinded observers. The highest rate of dye penetration in the contact surface with bleaching material was measured. In addition, the rate of dye penetration was scored according to the following classification: 0, without change; 1, light blue and 2, dark blue. The results of different cycles were compared [1].

***Statistical analysis***

Statistical analysis was performed using Statistical Package for Social Science (SPSS, version 20.0, Chicago, IL, USA). The Kendall coefficient was used to evaluate the inter-observer agreement. The chi-squared test was used to compare the scores of the samples. The level of significance was set at 0.05. 

## Results

**Figure 3:**
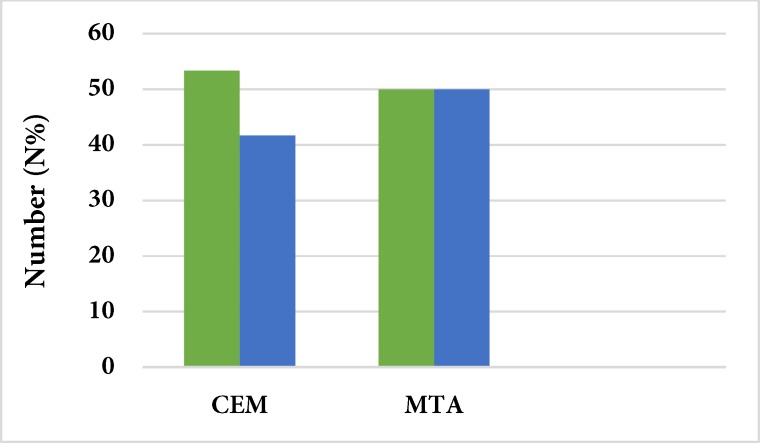
Comparing the sealing ability of MTA and CEM

The Kendall coefficient showed a relatively good inter-observer agreement (0.0735). Figures 1 and 2 describe the results of color changes of CEM and MTA as cervical barriers in comparison with negative and positive controls. The results of chi-squared test demonstrated that the rate of dye penetration of CEM and MTA were similar to that of positive control group, with no significant difference (*P*=0.673 and *P*=0.408, respectively). However, there was a significant difference between the negative control group and the CEM and MTA groups (*P*=0.001 for both groups). Figure 3 presents the results of comparison between MTA and CEM in terms of color change. The results showed that the sealing ability of MTA and CEM were the same, with no significant difference (*P*=0.682).

## Discussion

The placement of a cervical barrier in intra-coronal bleaching is an important and recommended treatment [3, 20-22]. The rationale behind this might be the isolating role of cervical barrier through preventing cervical resorption due to the penetration of peroxides from the pulp chamber into the periodontal ligament *via* the dentinal tubules [3, 23, 24]. The success of non-vital bleaching somehow depends on the sealing ability of the cervical barrier, especially when the remaining dentinal walls are very thin [25]. In addition, the significance of cervical barrier in leakage prevention was confirmed by Valadares *et al.* [26]. They concluded that the use of a cervical barrier prevents microleakage of *E. faecalis*. Studies are underway to determine an appropriate coronal barrier material with the best sealing properties.

In this *in vitro* study, we compared the sealing ability of CEM and MTA as a cervical barrier in intra-coronal bleaching. Recently MTA has been investigated for sealing the root canal [19]. The prevailing presence of calcium oxide in the formulation of MTA results in the release of calcium hydroxide during MTA hydration [27]. The role of calcium hydroxide in arresting or preventing tooth resorption has been demonstrated [28]. However, bleaching agents produce a low pH value on the root surface; this might be considered as a mechanism of action for cervical resorption [29]. Higher pH value of MTA and release of calcium hydroxide might further prevent cervical resorption.

Studies on CEM cement have indicated that it can release phosphorus and calcium ions which improve the alkalinity of environment and also lead to mineralization, suggesting its hard tissue inductivity [30]. CEM cement has already been evaluated for various applications, including treatment of furcal perforation, vital pulp therapies in permanent and primary teeth [30-39], root end filling [40], management of root resorption and pathologic/iatrogenic perforations [14, 41-43], periradicular surgery [42, 44] and revascularization of necrotic immature permanent molars [45]. Sealing ability of CEM cement has not been sufficiently evaluated, as a cervical barrier in intra-coronal bleaching. 

CEM cement has exhibited some advantages compared to MTA, such as shorter setting time and also significantly superior results in relation to film thickness and flow, easier handling, and enhanced antibacterial effects as well as better abilities to form hydroxyapatite in the presence of normal saline [13, 46]. Furthermore, favorable apical/coronal sealing property of CEM cement similar to that of commercial types of MTA and superior to Intermediate Restorative Material (IRM) has been shown in several studies [17, 42, 47, 48]. In addition, CEM cement is less costly in comparison to MTA [13].

The results of this study showed a significantly higher sealing ability of CEM cement compared to the negative control group, with no significant differences from the positive control group. Yavari *et al.* [49] showed better protection against microbial leakage of teeth with CEM cement in comparison with teeth without coronal seal.

The results of the present study showed that the sealing abilities of MTA and CEM were the same, with no significant differences. Our results are consistent with those reported by Yavari *et al.* [49] and Zarenejad *et al.* [50]. 

The results of this study showed the sealing ability of CEM cement as an intra-orifice plug to prevent penetration of dye, revealing no significant difference from MTA. This favorable sealing ability of CEM cement might be attributed to the reaction between calcium and phosphorous ions; however, it can also be related to the hydrophilic nature, favorable antibacterial/fungal potential, high pH value and formation of hydroxyapatite crystals [13, 46, 51-53].

## Conclusion

According to the results of this *in vitro* study, CEM cement can be used as a cervical barrier with sealing properties comparable to MTA during intra-coronal bleaching procedures.
